# The effect on tumour control probability of using AXB algorithm in replacement of AAA for SBRT of hepatocellular carcinoma located at lung–liver boundary region

**DOI:** 10.1259/bjro.20210041

**Published:** 2021-10-18

**Authors:** Michael Lok Man Cheung, Monica WK Kan, Vanessa TY Yeung, Darren MC Poon, Michael KM Kam, Louis KY Lee, Anthony TC Chan

**Affiliations:** ^1^ Department of Clinical Oncology, Prince of Wales Hospital, Hong Kong, SAR, China; ^2^ State Key Laboratory of Translational Oncology, The Chinese University of Hong Kong, Hong Kong, SAR, China

## Abstract

**Objective:**

To retrospectively analyze the clinical impact on stereotactic body radiation therapy (SBRT) for hepatocellular carcinoma (HCC) located at lung–liver boundary due to the use of Acuros XB algorithm (AXB) in replacement of anisotropic analytical algorithm (AAA).

**Methods:**

23 SBRT volumetric modulated arc therapy (VMAT) plans for HCC located at lung–liver boundary were calculated using AAA and AXB respectively with the same treatment parameters. The dose–volume data of the planned target volumes (PTVs) were compared. A published tumour control probability (TCP) model was used to calculate the effect of dosimetric difference between AAA and AXB on tumour control probability.

**Results:**

For dose calculated by AXB (Dose to medium), the D95% and D98% of the PTV were on average 2.4 and 3.1% less than that calculated by AAA. For dose calculated by AXB (dose to water), the D95% and D98% of the PTV were on average 1.8%, and 2.7% less than that calculated by AAA. Up to 5% difference in D95% and 8% difference in D98% were observed in the worst cases. The significant decrease in D95% calculated by AXB compared to AAA could result in a % decrease in 2 year TCP up to 8% in the worst case (from 46.8 to 42.9%).

**Conclusion:**

The difference in dose calculated by AAA and AXB could lead to significant difference in TCP for HCC SBRT located at lung–liver boundary region.

**Advances in knowledge:**

The difference in calculated dose and tumour control probability for HCC SBRT between AAA and AXB algorithm at lung–liver boundary region was compared.

## Introduction

Anisotropic analytical algorithm (AAA) is a commonly used dose calculation algorithm in radiation therapy treatment planning. It is a convolution-superposition algorithm taking lateral electron transport into account.^1,2^ Acuros XB algorithm (AXB) (Varian Medical Systems, Palo Alto, CA, USA) is a relatively new dose calculation algorithm which can accurately model dose deposition in heterogeneous media by solving linear Boltzmann transport equation with numerical methods.^1–3^ There are many publications on dose comparison between AAA and AXB for various tumour sites, such as lung,^1,2,4,5^ nasopharyngeal carcinoma,^6–8^ and breast.^9,10^ HCC located at the lung–liver interface is also very challenging, but literature on that was lacking. AAA, like other convolution-superposition algorithms, was shown to overestimate the dose at lung tissue interface for small fields of high energy photon beams.^11,12^ It uses the superposition of the Monte Carlo derived dose kernels of both primary and scattered components to obtain doses in voxels of the irradiated volume.^13^ However, the effect of tissue inhomogeneity is incorporated by simplified density scaling of the kernels such that the secondary electron transport is only modeled macroscopically. In this study, we would find out how this phenomenon affect the PTV dose coverage of HCC SBRT using volumetric modulated arc therapy (VMAT), and how the change in PTV dose coverage affect the tumour control probability (TCP) of HCC.

## Methods

### Patient selection and dose prescription

23 HCC SBRT patients with treatment site overlapping lung–liver boundary were selected for this retrospective analysis. The prescription dose followed Radiation Therapy Oncology Group (RTOG) 1112 clinical trial protocol, ranging from 27.5 to 50 Gy in 5 fractions, depending on the normal liver mean dose.^14^

### Treatment planning

The motion management for the patients was active breathing control or real-time monitored breath-hold. The planning target volume (PTV) was generated by adding 5 mm margin to the ITV in all directions. The average PTV volume was 164 cc (range: 15.4–664 cc). VMAT plans were created for the patients using 1–4 arcs with Millennium 120 multileaf collimator (MLC) (Varian Medical Systems, Palo Alto, CA, USA). The beam energy was 10 MV in flattening-filter-free (FFF) mode of a Truebeam (Varian Medical Systems, Palo Alto, CA, USA) linear accelerator, following the practice of using 10 MV beams for abdominal treatments, including HCC. This allowed a faster maximum dose rate of 2400 MU/min to be used when compared to 6MV-FFF (1400 MU/min).

The treatment plans were generated using treatment planning system Eclipse v. 13.6 (Varian Medical Systems, Palo Alto, CA, USA). Updated versions of this treatment planning system were available. The optimization criteria for the PTV followed RTOG 1112 protocol. At least 95% volume of the PTV received the prescription dose. The maximum dose allowed within the PTV was 150% of the prescription dose and the maximum dose allowed outside the PTV was 120% of the prescription dose as stated in RTOG 1112. But for most of the treatment plans in our institution (Prince of Wales Hospital, Hong Kong), the maximum dose outside PTV was kept below 105%. The ratio of the prescription isodose volume to PTV volume was less than 1.5. The dose limits for organs at risk also followed RTOG 1112.

The volumetric dose of the treatment plans was first calculated using AAA v. 13.6.23 with inhomogeneity correction, reporting dose to water by default. The treatment plans were then recalculated using AXB v. 13.6.23 with identical treatment parameters such as monitor units, MLC and gantry angle settings. The dose was calculated using dose to medium (Dm) option and dose to water (Dw) option with inhomogeneity correction. For dose to medium option, the electron fluence calculated by the AXB transport was multiplied by a medium-based flux-to-dose response function.^15–18^ For dose to water option, the electron fluence was multiplied by a water-based flux-to-dose response function. Dm could be rescaled to Dw using the ratio between the medium-based flux-to-dose response function and the water-based flux-to-dose response function, similar to stopping power ratio between medium and water used by Monte Carlo methods.^6^ The dose grid resolution was 2.5 mm for both AAA and AXB dose calculations.

### Dosimetric evaluation

**Figure 1.**
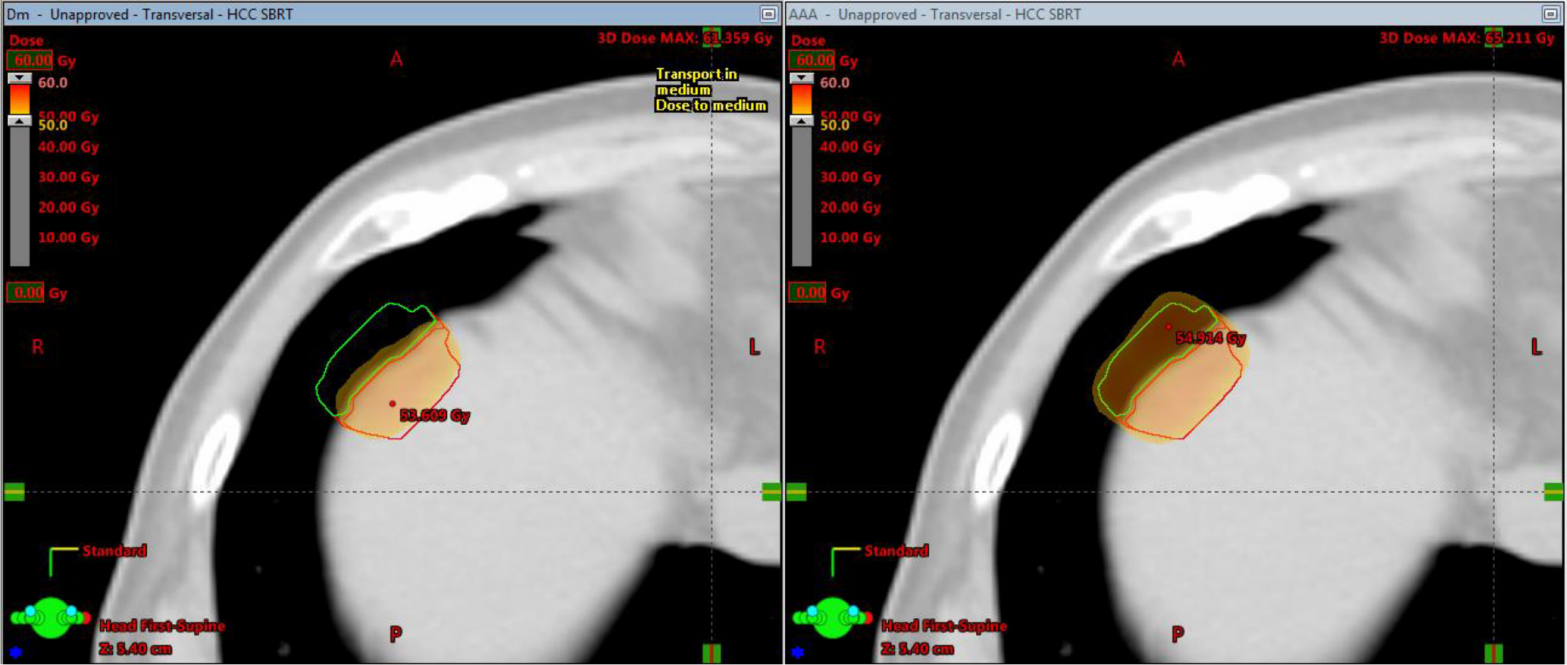
Dose comparison between AXB (Dm) and AAA. The PTV of HCC SBRT patient was split into two parts: The green contour was PTV_Lung. The red contour was PTV_SoftTissue. Notice the underdose region in PTV_Lung calculated by AXB (Dm) (left) compared to that calculated by AAA (right). [AAA, anisotropic analytical algorithm; AXB, Acuros XB algorithm; HCC, hepatocellular carcinoma; PTV, planning target volume; SBRT, stereotactic body radiation therapy]

In order to evaluate the dosimetric difference between AXB and AAA to the PTV due to inhomogeneity of medium, the whole PTV (PTV_Whole) of the patients were split into two parts, one part inside the lung tissue (PTV_Lung); the other one inside the soft tissue (PTV_SoftTissue) (Figure 1). The mean dose, D2% (dose received by 2% of PTV volume) and D98% (dose received by 98% of PTV volume) of the PTVs calculated using AAA, AXB (Dm) and AXB (Dw) respectively were compared. D95% was also included for PTV_Whole. Wilcoxon signed-rank test was used for dose comparison. The test was two-sided and *p*-values < 0.05 were considered statistically significant. The test was performed using SPSS Statistics 17.0 (SPSS Inc, Chicago, IL, USA).

### Tumour control probability analysis

The dose–volume histograms of the PTVs were converted to biologically effective dose (BED) using the linear quadratic (LQ) model conversion,^19,20^ assuming the α/β ratio to be 10, before being used for TCP calculation. A TCP model for 2 year TCP estimation of HCC SBRT fitted by Jang et al^21^ was used to evaluate the clinical implication of the dose differences between AAA and AXB. The TCP model was a logistic model in the following form:

(1) $$TCP=\frac{1}{1+(\frac{D_{50}}{D}{)}^{4/\gamma }}$$

Where D_50_ was the BED that led to 50% tumour control probability, D was the prescription dose to the tumour and γ was a parameter that controlled the slope of the TCP curve. For the Jang et al model, γ was 1.22. D_50_ was 34.9 Gy in three fractions, which corresponded to a BED of 75.5 Gy using LQ model conversion, assuming α/β ratio to be 10. Jang et al did not define exactly what the prescription dose D in the TCP model should be. In this study, we assumed D95% of the whole PTV to be the prescription dose D.

## Results

### Differences in dose to planning target volumes

The comparison between dose parameters of PTVs calculated by AAA and AXB was shown in Table 1. For dose to medium (Dm) calculated by AXB, the average deviation from AAA for the mean dose, D2% and D98% of PTV_SoftTissue were 1.4%, 1.8%, 1.9% respectively. On the other hand, the average difference in mean dose, D2% and D98% of PTV_Lung were 2.4%, 0.5%, 5.8% respectively. For PTV_Whole, the average difference in mean dose and D2% were similar to that of PTV_SoftTissue, while a larger average difference (3.1%) was observed for D98%, which was mainly contributed by the large dose difference in PTV_Lung. In the worst case, the difference observed in D98% for PTV_Whole could be up to 8%. A similar trend was observed for dose to water (Dw) calculated by AXB except that the average difference was slightly smaller for PTV_SoftTissue but slightly larger for PTV_Lung. The average difference in D95% between AAA and AXB was 2.4% for Dm and 1.8% for Dw. The largest difference in D95% was 5.1% for Dm and 5.4% for Dw. All the average dose differences between AAA and AXB were statistically significant (*p* < 0.05).

**Table 1.** Dose comparison for PTVs

		AXB (Dm) /AAA		AXB (Dw) /AAA	
PTVs	Parameters	%	p	%	p
PTV_SoftTissue	Dmean	98.5 ± 0.2	<0.001	99.8 ± 0.2	0.001
	D2%	98.2 ± 0.4	<0.001	99.6 ± 0.4	0.001
	D98%	98.1 ± 0.5	<0.001	99.3 ± 0.4	<0.001
					
PTV_Lung	Dmean	97.6 ± 1.9	<0.001	96.9 ± 1.7	<0.001
	D2%	99.5 ± 1.0	0.045	98.6 ± 1.0	<0.001
	D98%	94.2 ± 4.7	<0.001	93.8 ± 4.4	<0.001
					
PTV_Whole	Dmean	98.4 ± 0.2	<0.001	99.4 ± 0.3	<0.001
	D2%	98.3 ± 0.4	<0.001	99.7 ± 0.4	0.001
	D98%	96.9 ± 1.6	<0.001	97.3 ± 2.1	<0.001
	D95%	97.6 ± 0.9	<0.001	98.2 ± 1.3	<0.001

AXB, Acuros XB; PTV, planning target volume.

### Differences in tumour control probability

**Figure 2.**
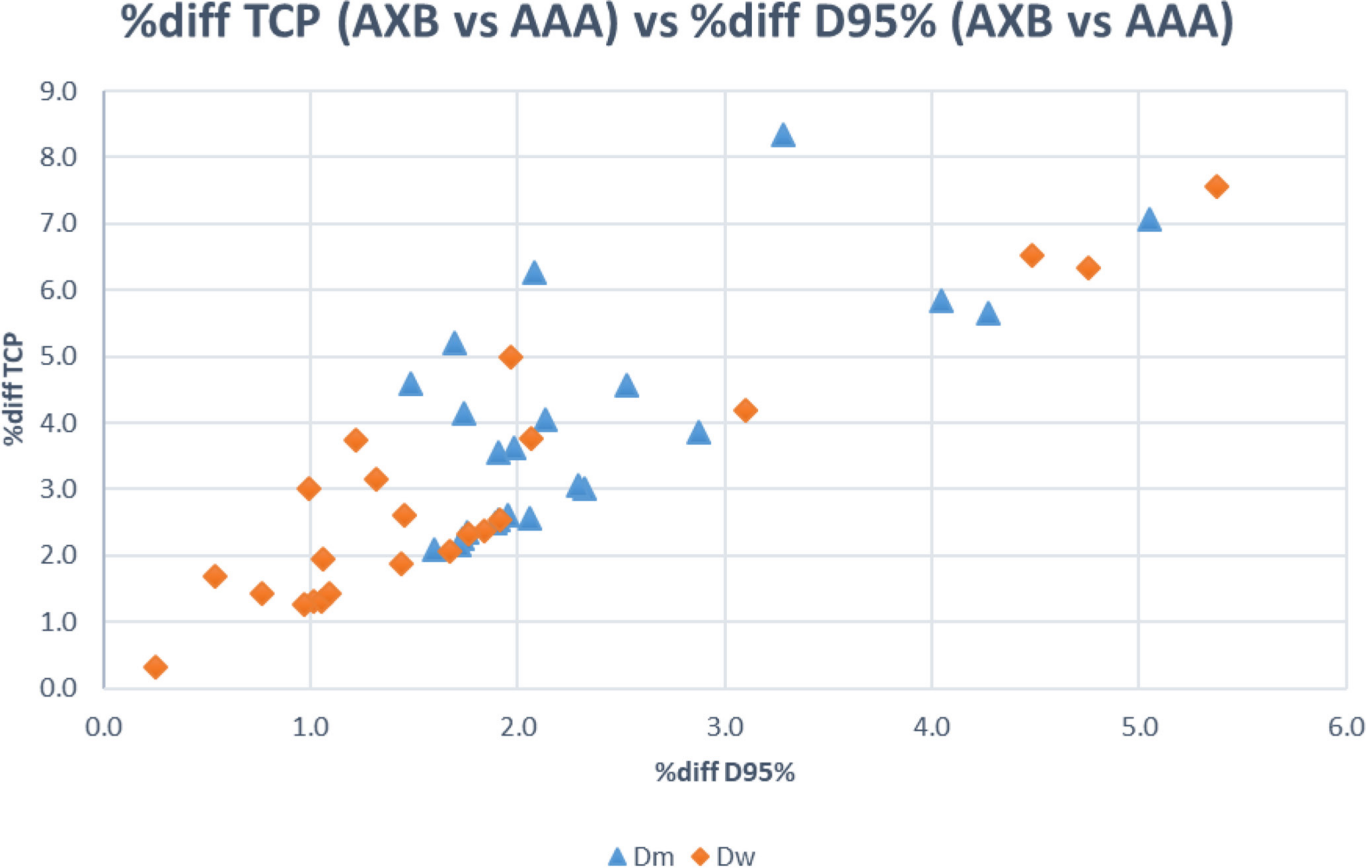
Percentage difference in TCP (AXB *vs* AAA) *vs* percentage difference in D95% (AXB *vs* AAA). [AAA, anisotropic analytical algorithm; AXB, Acuros XB algorithm; TCP, tumour control probability]

The median TCP was 73.8% for AAA, compared to 70.8% for Dm and 70.3% for Dw using AXB. The % difference in TCP due to the difference in D95% calculated by AAA and AXB was shown in Figure 2. The largest % difference in TCP was 8.3% for Dm and 7.6% for Dw. The mean % difference in TCP was 4.0% for Dm and 2.9% for Dw.

## Discussion

Our study showed that the dose difference in the mean dose and D2% of PTV_Whole between AAA and AXB was small, in general between 1–2% for Dm and 0.5–1.5% for Dw. But a larger difference was observed for D95% and D98%. For D95%, the average difference was around 2% on average and could be up to 5% in some cases. For D98%, the average difference was around 3% on average and could be up to 8% in some cases. The separate dose analysis for PTV_Lung and PTV_SoftTissue in our study confirmed that the decrease in D95% and D98% of the whole PTV was mainly due to the dose decrease in lung tissue calculated by AXB. This was in line with the results in the literature.^16–18,22^ This decrease in D95% calculated by AXB could lead to an average decrease in TCP of 4% for Dm and 3% for Dw. The largest difference could be up to 8% in some cases. This implied that the choice between AAA and AXB in dose calculation for HCC SBRT at lung–liver boundary region could be clinically significant.

The difference in calculated dose between AAA and AXB was mainly due to the inferior modelling of electron transport by AAA compared to AXB, especially for electron disequilibrium conditions in low density materials such as lung tissue for small field size. On the other hand, the dose difference between AXB (Dw) and AXB (Dm) was mainly due to the difference in the flux-to-dose response function, which was similar to stopping power ratio used for Monte Carlo methods between water, lung and muscle. For AXB (Dw), the water-based flux-to-dose response function was assigned for both lung tissue and soft tissue. For AXB (Dm), the lung-based flux-to-dose response function was assigned to lung tissue, while the muscle-based flux-to-dose response function was assigned to soft tissue. In our study, the average difference in AXB calculated mean dose reported by Dm and Dw in lung tissue was about +0.7%, and that in soft tissue was −1.3%, which were similar to the stopping power ratio found by Siebers et al for 10 MV photon beams (+0.8% between lung and water and −1.0% between ICRU tissue and water).^23^

Evaluation of the effect in TCP due to difference in calculated dose between AAA and AXB had been extensively conducted over the years. Petillion et al reported a decrease in TCP of 0.3% using AXB (Dm) and 1.1% using AXB (Dw) compared to AAA for breast radiotherapy.^24^ Padmanaban et al reported 1.2–3.1% decrease in TCP using AXB (Dm) for oesophageal cancer 3DCRT and VMAT.^25^ Liang et al reported a decrease in TCP up to 5.8% using AXB (Dm) compared to AAA for non-small-cell lung cancer SBRT.^22^ And more recently, Bufacchi et al found a decrease in TCP up to 6.8% using AXB (Dm) compared to AAA for nasopharyngeal carcinoma.^26^ The decrease in TCP was mainly due to a decrease in PTV dose coverage, which was more significant in region of lower density such as lung and air because of the difference in ability to model the electron disequilibrium in low density materials. The results of our study are basically in line with the literature, although the difference in TCP we found was a bit larger than that in the literature. The reason might be the use of 10MV-FFF beams in our study, compared to the more commonly used 6MV beams in the literature. It had been shown that higher energy beams would result in a more significant dose difference between AXB and AAA in low density regions.^15–18^ HCC was commonly considered as an abdominal treatment site. Using 10 MV beams instead of 6 MV beams could increase the penetration ability of the beam and for Varian Truebeam linacs, 10MV-FFF beams had a dose rate of 2400 MU/min, which is much faster than 1400 MU/min for 6 MV-FFF beams. But our study showed that for HCC at lung–liver boundary region, the benefits of 10 MV-FFF beams might be out-weighted by the clinical impact of decrease in TCP due to the significant decrease in dose coverage in the lung region of the PTV. Future research could be conducted to quantify the radiobiological impact of using different beam energy for HCC SBRT.

It should be noticed that the TCP model used in our study was obtained from another study based on different treatment techniques and dose calculation algorithms from the present study. Also, BED conversion in our study was based on LQ model, which might not be able to precisely predict the radiobiological effect of SBRT due to uncertainty in its robustness under high dose per fraction circumstances according to some studies.^27–29^ Therefore, the results in our study was only intended for relative comparison between AXB and AAA rather than finding the absolute TCP.

## Conclusion

The dose of SBRT for HCC located at lung–liver boundary calculated by AXB was in general less than that of AAA. The dose difference was more significant in lung tissue than in soft tissue, which could result in up to 5% decrease in D95% and 8% decrease in D98% of the PTV calculated by AXB when compared to AAA. The significant decrease in D95% could result in a decrease in 2 year TCP up to 8%.
